# Facial erythema detects diabetic neuropathy using the fusion of machine learning, random matrix theory and self organized criticality

**DOI:** 10.1038/s41598-020-73744-3

**Published:** 2020-10-08

**Authors:** Esmaeil S. Nadimi, Tomas Majtner, Knud B. Yderstraede, Victoria Blanes-Vidal

**Affiliations:** 1 Applied AI and Data Science (AID), Maersk Mc-Kinney Moller Institute, University of Southern Denmark, 5230 Odense, Denmark; 2 Steno Diabetes Center Odense, Odense University Hospital, 5230 Odense, Denmark

**Keywords:** Endocrinology, Engineering, Electrical and electronic engineering

## Abstract

Rubeosis faciei diabeticorum, caused by microangiopathy and characterized by a chronic facial erythema, is associated with diabetic neuropathy. In clinical practice, facial erythema of patients with diabetes is evaluated based on subjective observations of visible redness, which often goes unnoticed leading to microangiopathic complications. To address this major shortcoming, we designed a contactless, non-invasive diagnostic point-of-care-device (POCD) consisting of a digital camera and a screen. Our solution relies on (1) recording videos of subject’s face (2) applying Eulerian video magnification to videos to reveal important subtle color changes in subject’s skin that fall outside human visual limits (3) obtaining spatio-temporal tensor expression profile of these variations (4) studying empirical spectral density (ESD) function of the largest eigenvalues of the tensors using random matrix theory (5) quantifying ESD functions by modeling the tails and decay rates using power law in systems exhibiting self-organized-criticality and (6) designing an optimal ensemble of learners to classify subjects into those with diabetic neuropathy and those of a control group. By analyzing a short video, we obtained a sensitivity of 100% in detecting subjects diagnosed with diabetic neuropathy. Our POCD paves the way towards the development of an inexpensive home-based solution for early detection of diabetic neuropathy and its associated complications.

## Introduction

Diabetes is a major health issue that has reached alarming levels. In 2019, nearly 9.3% of adults 20–79 years are living with diabetes worldwide. The estimated number of people (20–79 years) living with diabetes has increased by 62% during the past 10 years. The number is projected to increase by 25% in 2030 and 51% in 2045^1^.

Diabetic neuropathy (DN), a type of nerve damage caused by long-term elevated glucose levels, is the most common complication of both type 1 and 2 diabetes and occurs in more than half of affected individuals. The condition usually develops slowly and sometimes over the course of several decades. Many studies have shown a significant negative impact on quality of life of those diagnosed with DN^2^. Furthermore, a substantially increased mortality among individuals diagnosed with diabetes peripheral neuropathy (DPN) who have undergone a major amputation, with 5-year mortality ranging from 44 to 68% has been observed. This calls for urgent action towards addressing this growing global health problem^3^.

To a high degree, most complications associated with different types of DN (such as amputation, digestion problems, double and impaired vision, among others) could be prevented through multidisciplinary care, which not only reduces complication risk, but also substantially reduces the rates of hospitalisation and recurrence^4^. Therefore, an early clinical assessment of DN is essential, despite the fact that the diagnosis of DN can be time consuming, costly and invasive. To address these shortcomings, innovative point-of-care devices (POCDs) play a fundamental role in developing innovative frameworks for screening and early multi-factorial intervention as the best prospect for preventing or halting the progress of DN and its devastating complications and sequel. POCDs firstly enable early diagnosis of DN and secondly, assess the evidence for early management strategies based on medication, dietary and lifestyle changes to reduce the incidence and slow the progression of these complications^5^. Examples of such POCDs include, but is not limited to, DPNCheck^6^, Neuropad^7^ and Sudoscan^8^ that rely on electromyography, measurements of Sural nerve conduction velocity and response amplitude as standard bio-markers for diagnosis of asymptomatic and clinical DPN. Further techniques rely on cerebrospinal fluid analysis, nerve biopsy findings and infrared pupillography (for evaluation of Argyll-Robertson pupil), often associated with autonomic neuropathy^9^. A recent novel non-invasive technique based on corneal confocal microscopy to quantify small fibre pathology in peripheral neuropathies and to provide in-vivo images of corneal nerve fibres was introduced by^10^.

Rubeosis faciei, characterized by a chronic facial erythema (reddening of the face), is associated with non-cutaneous diabetic complications including nephropathy, retinopathy and neuropathy^11^^,^^12^. This is possibly due to sharing microangiopathy as a common etiological factor. The exact pathogenesis of rubeosis diabeticorum is unknown, and therefore, it may follow different pathogenic pathways in subgroups of patients. This might, at least in part, explain the heterogeneity in clinical presentation observed between individuals. In routine clinical practice, facial redness of patients with diabetes is evaluated based on subjective clinical observations of visible redness. If rubeosis faciei is recognized, it should alert physicians to look for other microangiopathic complications. However, rubeosis faciei may go unnoticed in routine clinical practice, since the intensity of red coloration depends on the degree of vascular engorgement of the superficial venous plexus, as well as on the skin tone^11^. On the contrary, if facial erythema is detected, it can be mistaken for other dermatological conditions. Recently^13^, deployed Mexamater MX18 (Courage & Khazaka electronic GmbH—Cologne, Germany)^14^ and AGE Reader (DiagnOptics Technologies B.V.—Groningen, Netherlands)^15^ to assess two components responsible for skin color, namely melanin and haemoglobin, the later being associated with erythema, and further estimate the immediate cardiovascular risk.

Mexamater MX18 quantifies erythema based on the principle of absorption and reflection of two specific wavelengths, i.e., green (568 nm) and red (660 nm), corresponding to the spectral absorption peak of haemoglobin while avoiding other colour influences (e.g. bilirubin). These measurements are then quantified in terms of erythema index, ranging from 0 to 999. Even though Mexamater MX18 was primarily designed for assessing skin condition in cosmetic applications, one can find a rich literature in deploying MX18 in other scientific disciplines^14^. The AGE Reader, on the other hand, is a non-invasive POCD that uses ultra-violet light to excite autofluorescence in skin tissue. The autofluorescence is from the level of Advanced Glycation End products (AGEs), providing an immediate cardiovascular risk prediction. The main weakness is that recent studies could not demonstrate a significant statistical correlation between facial erythema index and skin autofluorescence. Other shortcomings of AGE Reader are that it has not been validated for measurements on very dark skin tones, and an error message is displayed when unreliable measurements are collected^15^.

The main objective of this study is to design an inexpensive home-based, contactless, non-invasive diagnostic POCD for identification of patients with diabetes without diagnosed complications, showing the very early signs of peripheral or autonomic neuropathy, by monitoring for early signs of rubeosis faciei. Our POCD consists of a digital camera for recording faces of subjects, for further magnification of spatio-temporal variations of subtle changes in the skin color along with motion in saccadic movements and deformation in pupil dilatation. Our hypothesis is twofold: (1) the recorded video carries important imperceptible and subtle variations in subject’s skin color that fall below humans’ limited spatio-temporal sensitivity. By revealing these variations using image processing, and estimating the time series associated with the difference between highest and lowest color intensity, spatio-temporal tensor expression profile for color variations caused by subject’s pulse can be obtained. Some feature characteristics of these tensor expression profiles for diabetic subjects will differ from those of a control group. (2) By tracking subject’s iris and pupil movements and deformations, and after canceling involuntary head movements out (caused by subjects pulse and respiration), spatio-temporal expression profile for saccadic movements are obtained. Similar to our first hypothesis, we believe that some feature characteristics of these tensor profiles differ between diabetics and control group. In this paper, we will address the first hypothesis. The second hypothesis is thoroughly discussed in^16^ and we therefore refer interested readers to that study.

The organization of this paper is as follows. In "Research design and experimental set-up" section, we present study participants and the experimental setup to acquire the videos of diabetic and control subjects. In "Proposed methodology" section, processing of retrieved videos by applying Eulerian Video Magnification (EVM) technique to reveal subtle changes in color variations of subject’s skin, and to further estimate the time series associated with these variations are presented. We further touch base concepts around the distribution of eigenvalues linked to the theory of random matrices (RMT), and processes exhibiting power law linked to Self-Organized-Criticallity (SOC). Having found some discriminatory features, we will cruise through the most important algorithms based on optimal ensemble learning for classification purposes. In "Results" section, we apply these concepts to the time series associated with the spatio-temporal tensor expression profile for color variations obtained by EVM among both diabetic and control subjects. Conclusions, discussions and future works are provided at the end of this paper.

## Research design and experimental set-up

### Study participants

The study enrolled 27 subjects, divided into two groups. Group 1, hereafter referred to as DM (Diabetic Mellitus) included 18 patients with diabetes (age: ${63 \pm 10}$ years; 12 males and 6 females with BMI of ${29 \pm 7}$). Group 2, hereafter referred to as C (Control) included 9 control subjects (age: ${60 \pm 6}$; 6 males and 3 females of with BMI of ${28 \pm 4}$). The two groups did not differ in average age, gender proportion or BMI ($p>0.10$). 10 DM patients were diagnosed with type 1 diabetes (time from diagnosis: ${28 \pm 13}$ years), while remaining 8 patients were diagnosed with type 2 DM (time from diagnosis: ${22 \pm 5}$ years)^11^.

Out of the 18 DM patients, 17 had been diagnosed with peripheral neuropathy, 17 with retinopathy, and 7 with diabetic nephropathy, and 5 of them had been diagnosed with peripheral arterial disease. The diagnosis of neuropathy was carried out using Semmes–Weinstein monofilament 10 g and biothesiometry indicating neuropathy with a threshold of 25 V. Facial erythema was not apparent in any of the participants, except for one DM patient with slight facial red coloration. None of the DM patients had exhibited cranial neuropathies such as facial nerve palsy, optic neuropathy, or auditory neuropathy. Furthermore, no individual was diagnosed with obstructive sleep apnoea (OSA). More information on DM patients can be found in Table 1. Control subjects had not been diagnosed with DM, peripheral arterial disease, or any disease affecting the nervous system.

The study protocol was reviewed and approved by the scientific ethical committee for Region Southern Denmark (process number: 18/297; Project ID: S-20180006). All participants were informed about the study, and signed, written informed consents to publish the results of this study were obtained. Furthermore, informed consent for participation was obtained from the participants in the manuscript. All methods and experiments were carried out in accordance with relevant guidelines and regulations based on the Declaration of Helsinki.

**Table 1:** Baseline characteristics of patients with diabetes.

Type of diabetes	Neuropathy	Retinopathy	Gender	Age	Duration of diabetes (years)	Duration of microvascular complications (years)
1	Peripheral	Yes, non-proliferative	F	50	31	> 15
			F	56	48	> 15
			M	49	18	4
			M	58	28	> 15
			M	65	16	12
			M	70	32	8
			M	73	15	> 15
		Yes, proliferative	M	72	36	> 15
			M	65	51	> 15
	Autonomic		M	30	21	11
2	Peripheral	No	F	72	24	2
		Yes, non-proliferative	F	70	30	>15
			M	64	23	9
			M	67	20	12
			M	71	22	> 15
		Yes, proliferative	F	60	26	> 15
			F	63	11	11
			M	67	19	> 15

### Experimental set-up

The experimental set-up included a laptop and a Canon EOS 1300D digital camera mounted on a tripod. To conduct the experiment in a realistic set-up, subject’s illumination was not directly controlled, and to compensate for that, colour constancy methods were deployed. The most important aspects to be considered in pursuance of the acquisition of an input video of adequate quality for subsequent processing were as follows: (1) stable camera, (2) constant background, (3) minimal reflections and shadows, (4) non-moving objects within the frequency range of interest, (5) choice of a digital camera, (6) sufficient temporal resolution of the video (frame rate), (7) sufficient spatial resolution of the video and color depth, (8) short distance to the object and optics, (9) proper angle of the view point and (10) sufficient duration of the video^17^.

**Figure 1:**
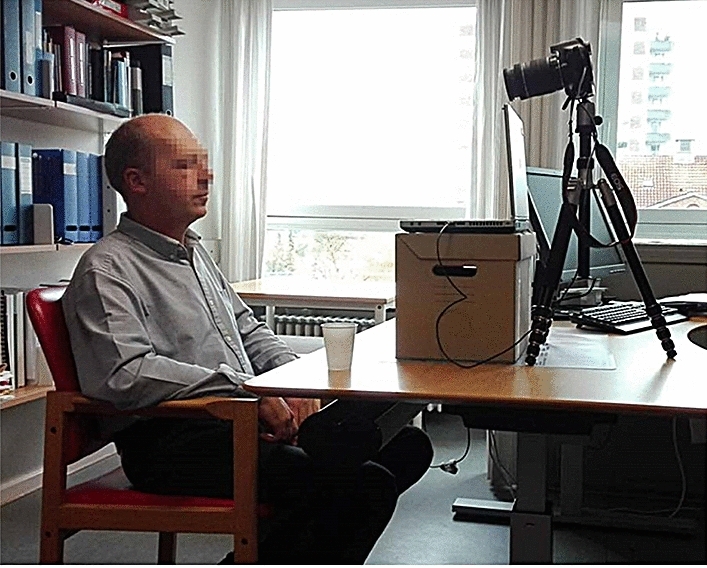
Experimental set-up.

The camera was placed above the laptop, about 84 cm in front of the subject being examined, so that the entire face was captured. The subject was seated on a comfortable chair while looking at the laptop’s screen, see Fig. 1. The subject was then asked to follow with their eyes a target on the screen, with the shape of a white circle moving on a black background either in a sudden form (to force saccadic movements), or in a smooth fashion (to force pursuit motions), while keeping the head as still as possible. A series of movements occured randomly over a period of 7 min and 45 s (including two sets of 30 s breaks). Before each command, a sound cue was emitted, to facilitate the synchronization between the video and the commands. Two repetitions were recorded on each participant, with a resting time between repetitions of about 10 min. In all cases, the camera was set to record with a focal length of 35 mm, at 50 frames per second (fps), and a frame size of 1280 $\times$ 720 pixels.

Once the pre-processing of subject’s videos was performed, including trimming the unwanted parts of the video footage, i.e., those parts that could result in inaccurate measurements of color intensity, such as the breaks in which subjects could abruptly move their head or turn away from the camera, six video segments of 5,901, 4,851, 6,251, 5,901, 4,851 and 6,251 frames each (approximately 2 min long), were collected. These videos were then fed into EVM for magnification of subtle changes invisible to the naked eyes.

It is worth noting that two factors that could affect facial erythema during video acquisition and potentially introduce errors were compensated for: (1) individual-specific factors that mostly affect facial color in a homogeneous manner, equally affecting all regions of the face (e.g. individual-specific score in the Fitzpatrick scale, sun exposure if bilateral) and (2) circumstantial factors that cause color differences among different facial regions (e.g. physical activity, emotional state, and certain skin conditions)^18–20^. We addressed the first potential source of error by using the difference of redness between two different face regions to begin with. We minimized heterogeneous color variations caused by the second potential source of error by using the same protocol for all participants, for example, by ensuring that none of participants engaged in any physical activity during the previous twenty minutes prior to the beginning of experimental recordings.

## Proposed methodology

**Figure 2:**
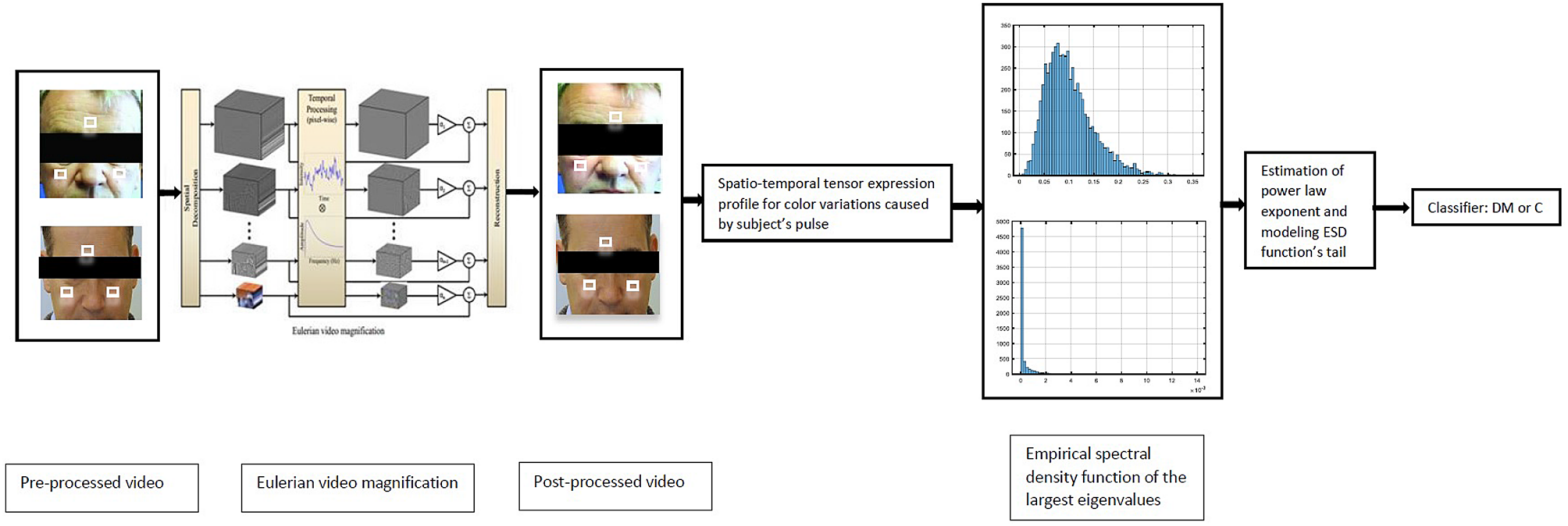
Schematic of our proposed method.

Our method relies on the fusion of information obtained from (1) applying EVM to the videos, (2) studying empirical spectral density (ESD) function of the largest eigenvalues of our tensors obtained from EVM using theories of random matrices, (3) quantifying these ESD functions and modeling their tails in terms of Generalized Pareto distribution and power law exponents $f(x)=Cx^{-\gamma }$ in systems exhibiting SOC, and (4) designing an ensemble learning-based classifier to split the subspace corresponding to group DM from that of group C. Schematic of our proposed method is shown in Fig. 2. The future predictive performance of the method was estimated using Leave-one-out Cross-Validation (LOO-XV) technique.

### Eulerian video magnification (EVM)

Revealing important subtle changes that fall outside human visual limits to be seen with the naked eye, and displaying them in an indicative manner using Eulerian video magnification technique was first introduced by^21^. EVM takes a video as input, and applies a sequence of spatial decomposition and temporal filtering to the frames. The filtered outcome is further amplified by a magnification factor $\alpha$ to reveal hidden information such as color variations or head movements caused by subject’s pulse. This technique can show events of interest that occur at specific temporal frequencies, such as the frequency band corresponding to the human pulse. The main advantages of EVM compared to the Lagrangian video magnification or other similar techniques are (1) its capability to cope with dynamic environments, (2) its robustness under different noisy condition and (3) its invariant performance under different skin-tones.

Assume image intensity function at pixel *x*, *y* at time *t* be determined by *I*(*x*, *y*, *t*). After translational variation, lasting *t* units of time, the initial image intensity function $I(x,y,t=0)=f(x,y)$ evolves into $I(x,y,t)=f(x+\delta (x),y+\delta (y),t+\delta (t))$, where $\delta (.)$ is the displacement function and therefore, magnified intensity function is calculated as follows:

1 $$\begin{aligned} {\hat{I}}(x,y,t)\approx f(x,y,t) + (1+\alpha ) \delta (t) \frac{\partial ^2 f(x,y)}{\partial x \partial y} \end{aligned}$$

where $\alpha$ is the magnification factor. To increase temporal signal-to-noise ratio and for the sake of computational efficiency, pooling multiple pixels while simultaneously processing spatially using low-pass filters were applied to the frames of the video. In this study, however, we benefited from full Laplacian pyramid spatial filtering while for the temporal processing, subtraction of two Butterworth low-pass filters with cutoff frequencies of 4.0 and 0.4 Hz was selected. The extracted band-pass signal was then multiplied by a magnification factor, namely $\alpha$ = 50. The temporal processing was uniform for all spatial levels and for all pixels within each level. The filtered spatial bands were then added back to the original signal and collapsed to generate the output video with magnified color. We used the post-processed videos and automatically extracted eight facial patches of size $31 \times 31$ pixels from each frame, featuring two patches corresponding to two areas potentially affected by facial erythema (i.e., cheeks) and six patches corresponding to facial areas less prone to erythema, representing the background facial skin tone (forehead, philtrum, and nose). The location of the patches is detected all through the video, using an algorithm for the detection of facial landmarks. The intensity value of the Red channel in the RGB color spectrum of each $31 \times 31$-pixel patch was the basis for forming the spatio-temporal tensor expression profile for color variations. The difference between the highest and lowest intensity patches estimates normalized spatio-temporal tensor expression profile for color variations caused by subject’s pulse.

### Random matrix theory (RMT)

RMT attempts to make statements about the statistics of the eigenvalues $\lambda _{\eta }$ of large random matrices, in particular the density of eigenvalues $\rho (\lambda )$ defined as below:

2 $$\begin{aligned} \rho _N(\lambda )=\frac{1}{N} \sum _{\eta =1}^{N} \delta (\lambda -\lambda _{\eta }) \end{aligned}$$

where $\lambda _{\eta }$ are the eigenvalues of the N $\times$ N symmetric matrix *H* that belongs to the statistical ensemble under scrutiny, and $\delta$ is the Dirac function. The Marcenko–Pastur theorem^22^ on the spectrum of empirical correlation matrices have been implemented in many, very different contexts including neural networks, image processing and population health studies. The question raised is the feasibility to identify common causes that explain the dynamics of various quantities linked to $\lambda _{\eta }$. These quantities might resemble the motion of individual grains in a packed granular medium linked to systems featuring self-organized-criticality, or different biological indicators such as blood pressure or cholesterol level within a population^23^. Inspired by these studies, we attempted in this work to draw statements about the statistics of the eigenvalues of the spatio-temporal tensor expression profiles for the color variations using RMT. We looked at the statistics of the bulk of eigenvalues and deployed the covariance matrix model to make statements about the empirical spectral density (ESD) function of the largest eigenvalues of our tensors.

Tracy and Widom^22^ showed that upon following normalization of $\lambda _{max}$, cumulative distribution function (CDF) of $\xi$ could be estimated as follows:

3 $$\begin{aligned}{}& \xi \equiv \sqrt{2} N^{\frac{1}{6}}(\lambda _{max}-\sqrt{2N}) \nonumber \\& F(\xi ) = exp\left( -\int _{\xi }^{\infty } (x-\xi ) q^2(x) dx\right) \end{aligned}$$

where *q*(.) is the solution to the nonlinear differential equation associated with the Airy function. It is worth noting that the Tracy–Widom distribution can be as well estimated by a Gamma distribution, $\Gamma (\beta ,\theta )$, with following pdf

4 $$\begin{aligned} f(x;\beta ,\theta )=\frac{\theta ^{\beta }x^{\beta -1}exp(-\theta x)}{\Gamma (\beta )} \end{aligned}$$

where $x>0$ and both shape and rate parameters $\beta ,\theta$ are positive.

In this research, we study the statistics of the largest eigenvalues of the normalized spatio-temporal tensor expression profiles associated with each video segment (6 per subject for both group DM and C) to explore whether they are asymptotically governed by the Tracy–Widom distribution. We also pay special attention to the appearance of sharp edges and particularly to the tails of the spectrum and their decay rate.

### Generalized Pareto (GP) and $\Gamma$ distribution

The GP distribution, a generalization of both Exponential distribution and the Pareto distribution, is a right-skewed distribution, parameterized with location parameter $\mu$, a shape parameter *k*, also known as the tail index parameter, and a scale parameter $\sigma$. *k* can be positive, zero, or negative and was developed to model tails of a wide variety of distributions. The probability density function of $X\thicksim GPD(\mu , \sigma , k)$ is

5 $$\begin{aligned} f_{(\mu ,k,\sigma )}(x)=\frac{1}{\sigma }\left(1+k\frac{(x-\mu )}{\sigma }\right)^{(-1-\frac{1}{k})} \end{aligned}$$

in which $x\ge \mu$ when $k\ge 0$, and $\mu \le x \le \mu - \frac{\sigma }{k}$ when $k<0$.

Distributions whose tails fall off as a polynomial (i.e., power law), such as *Student’s t*, lead to a positive shape parameter *k*. Distributions whose tails decrease exponentially, such as the normal, correspond to a zero *k*, while distributions with finite tails, such as Gamma, correspond to a negative shape parameter *k*. We estimate the right tail index parameters *k* of ESD function of the largest eigenvalues derived from the normalized spatio-termporal tensor expression profiles of video segments for both groups DM and C by fitting GP distribution. This indicates whether a subject follows $k>0$, representing power law behaviour and therefore exhibiting SOC, or following $k<0$, representing Tracy–Widom distribution [estimated by $\Gamma$ in Eq. (4)] and therefore exhibiting a more unstructured random behavior.

### Self-organized-criticality (SOC)

Critical systems are dynamical systems with several interacting components that exhibit scale-invariant fluctuations^23^. Experiments have suggested that the healthy brain, capable of self-tuning to the critical state, also known as SOC, functions near phase transitions because criticality improves both information processing capabilities and health^23^. Experiments have shown that when the brain malfunctions, e.g., during epileptic seizures, the brain loses the characteristics of criticality. Experiments have also shown that the size of neural avalanches appears in various aspects of observables of the system, following power-law distributions of the form $f(\lambda _{max})=C\lambda _{max}^{-\gamma }$, which are generally a consequence of scale invariance and therefore an evidence for criticality^23^.

We estimate the exponent $\gamma$ following the commonly accepted Maximum Likelihood Estimator (MLE) which provably leads to accurate parameter estimates in the limit of relatively large sample size. The exponent with an error up to *O*(1/*N*), *N* being the sample size, can be estimated as follows:

6 $$\begin{aligned} {\hat{\gamma }}=1+N\left[ \sum _{i=1}^{N}\ln \left( \frac{x_i}{x_{min}}\right) \right] \end{aligned}$$

under the assumption that $\gamma > 1$, and $x_{min}$ represents the cutoff value for the tail of the distribution.

Reference ^23^ found that the avalanche size of neuronal tissues in a healthy brain follows a power law with exponent $\gamma$ close to $-1.5$, with the avalanche duration following a power law with an exponent close to $-2$.

### Ensemble learning-based classification

Combining several base predictive learners using an ensemble of models aims at providing better predictions due to capturing the underlying distribution of the data in a more precise manner^24^. Different ensemble-based techniques range from bagging to boosting and stacking have been used in different research disciplines including health^24^. While the first two techniques, namely bagging and boosting primarily focus on reducing either variance or bias, stacking approaches attempt at finding the optimal approach to accumulate base learners so that the best trade-off between bias and variance is obtained. Stacking technique searches for optimal weights using cross-validation, also known as Cross-validated Optimal Weighted Ensemble (COWE). COWE as presented in Table 2 intends to find the best way to combine predictions of base learners such as decision trees, linear discriminant analysis (LDA), Naive Bayes, support vector machines (SVM), K-nearest neighbors (KNN) and neural networks among others, by searching for the optimal weight to combine them so that the outcome (ensemble) minimizes the total expected prediction error (MSE). The optimization model of COWE assumes that the hyperparameters of each base learner are tuned prior to conducting the weighting task. This means that the hyperparameters are tuned in an optimal fashion as an independent process. The optimization process relies on three distinct algorithms, namely Bayesian optimization, random search or grid search. While the former aims at approximating the unknown function with surrogate models like Gaussian process, the two latter solutions are exhaustive search methods. Bayesian optimization tries to gather observations with the highest information in each iteration by making a trade-off between exploration and exploitation.

**Figure 3:**
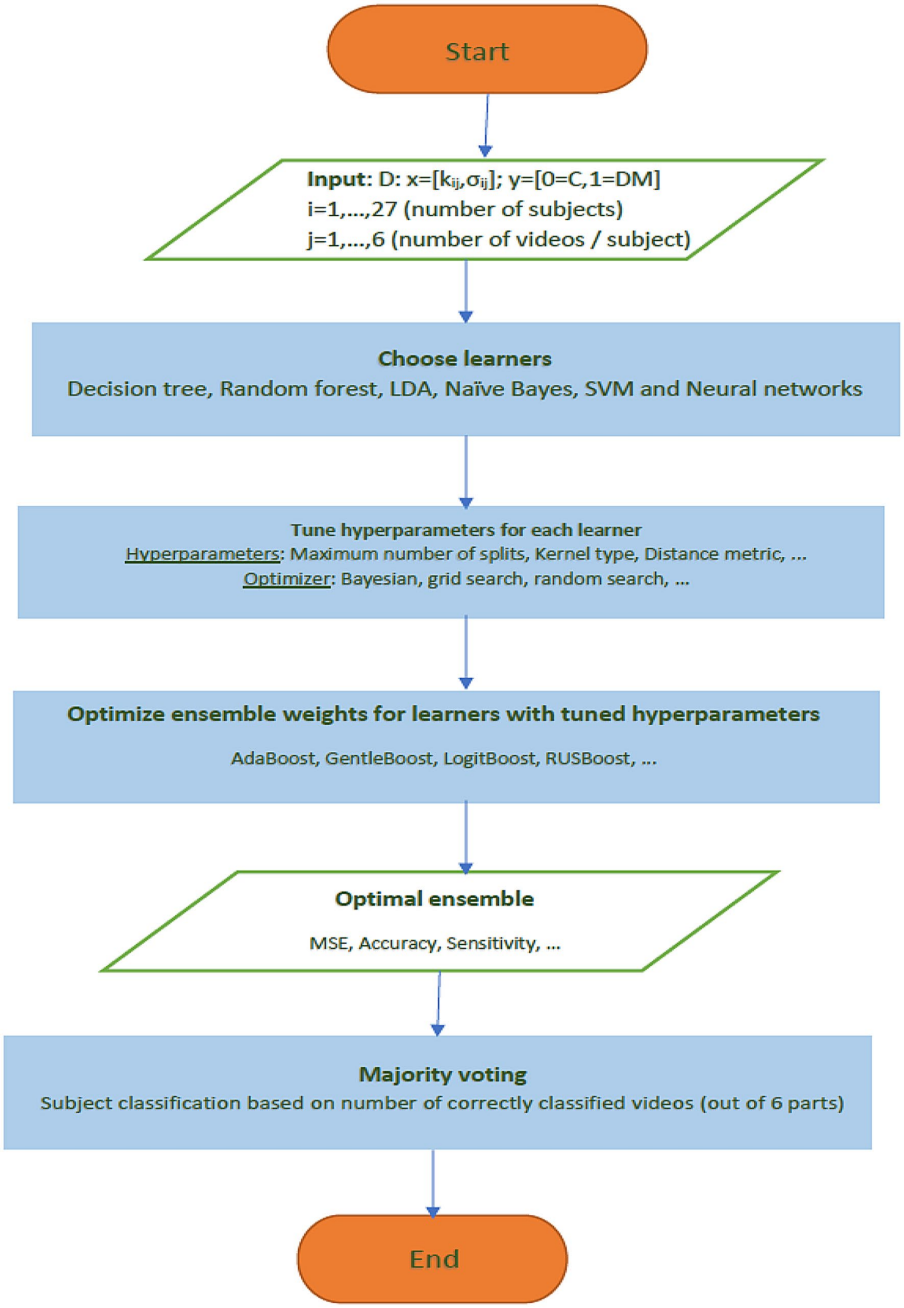
Architecture of our cross-validated optimal weighted ensemble (COWE)-based classifier.

The architecture of our classifier is presented in Fig. 3, in which *k* and $\sigma$ of fitted $GP(k,\sigma )$ to the right tail of ESD function of the largest eigenvalues of normalized spatio-temporal tensor expression profiles for color variations retrieved from subject’s 6 video segments are the feature predictor variables, while class C (0) and DM (1) are the response variables. A wide range of learners, their corresponding hyperparameters, and optimizers to search for them, along with the optimal ensemble weights were investigated.

We deployed Leave-one-out Cross-Validation (LOO-XV) to predict how well the developed method for feature extraction and classification will generalize on an independent data set. LOO-XV removes each observation in turn, constructs the classifier, and then computes whether this leave-one-out classifier correctly classifies the deleted observation. This was iterated 27 times (27-fold cross validation at subject level) with a different observation (subject) reserved for testing purpose each time. The final assignment of a subject belonging to either class was based on majority voting with equal weights, i.e., 4 equally classified video segments out of 6 (per patient) dictates the final class, where a tie is considered undecided. The performance of the classifier was finally calculated from the 27 testing observations, by using pre-determined performance metrics such as accuracy, sensitivity and specificity.

**Table 2:**
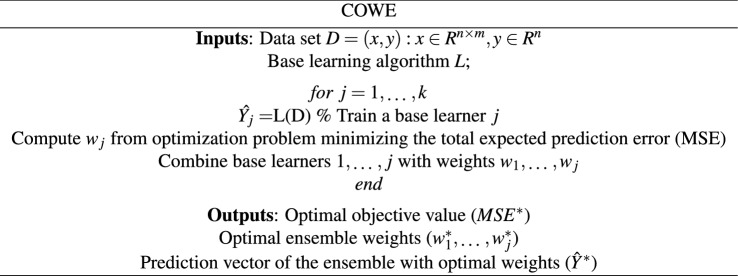
Cross-validated optimal weighted ensemble (COWE) algorithm.

## Results

### Application of EVM to subject’s videos

**Figure 4:**
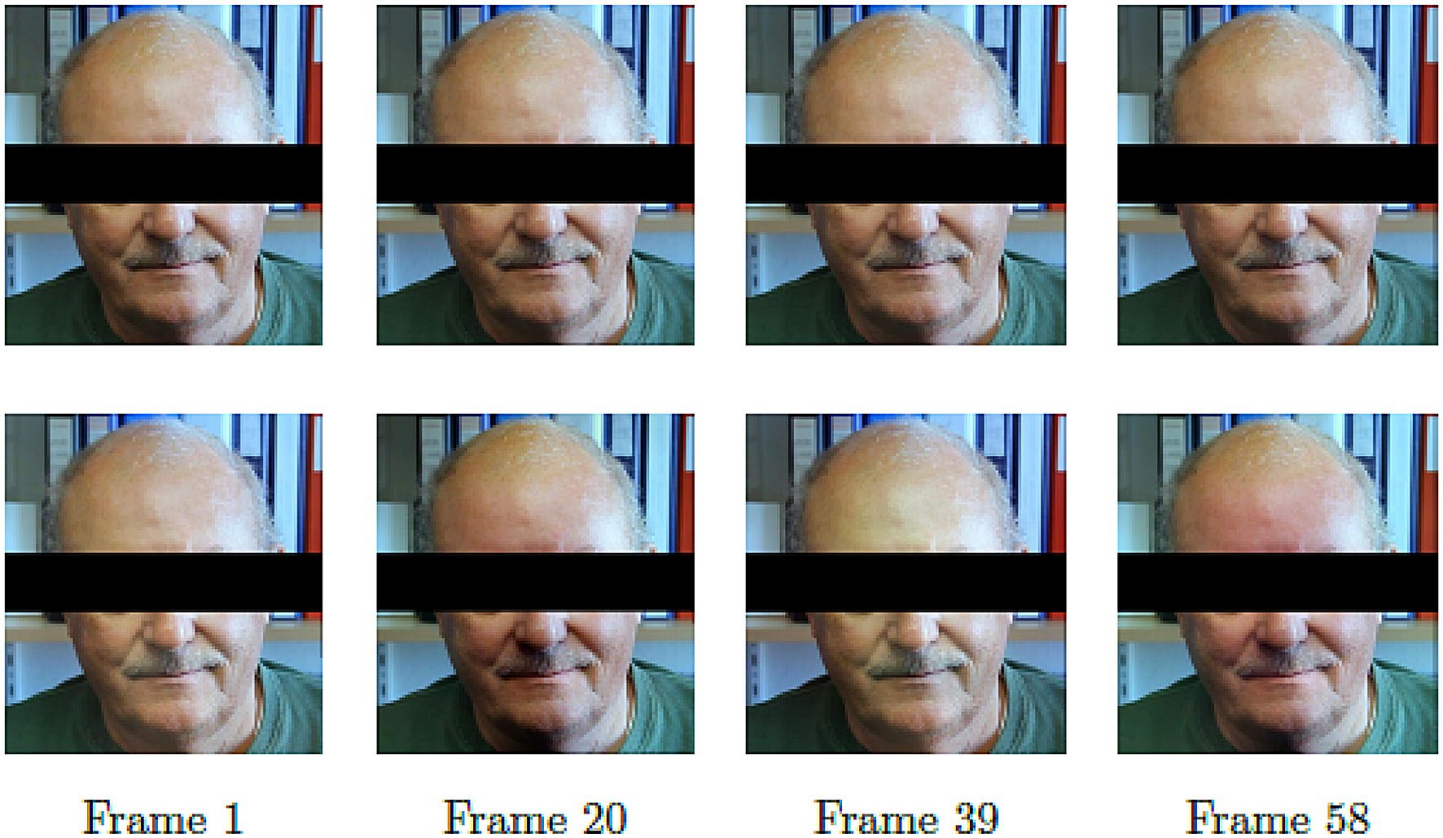
Illustration of temporal facial color variations before (top row) and after applying Eulerian Video Magnification (bottom row).

**Figure 5:**
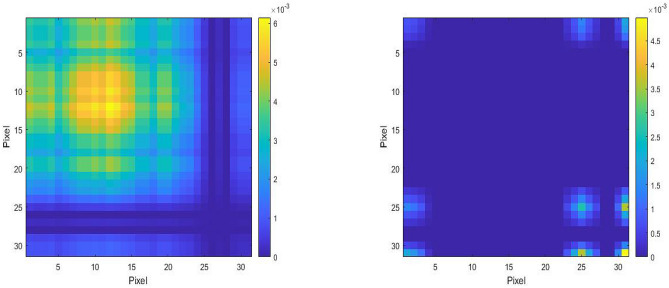
Example of the difference between the highest and lowest intensity value of the Red channel (RGB color spectrum) of a $31\times 31$-pixel patch. The left patch belongs to a subject from group DM and the right patch is generated from a subject of group C.

An illustration of temporal color variations of subject’s face due to the pulse, before (top row) and after applying EVM with magnification factor $\alpha =50$ (bottom row) is presented in Fig. 4. Subtle color changes invisible to the naked eye (top row) is evident after magnification (frames 0 and 39, vs. frames 20 and 58), where the time between heart beats is about 0.38 s (19 frames in a video at 50fps, bottom row). An example of the difference between the highest and lowest intensity value of the Red channel (RGB color spectrum) of each $31 \times 31$-pixel patch to estimate the spatio-temporal tensor expression profile for color variations after magnification is presented in Fig. 5. The intensity map on the left belongs to a subject from group DM, while the equivalent on the right belongs to a subject chosen from group C. The intensity maps show that the mean of intensity difference among subjects of group DM is approximately 22 times larger than that of group C.

### Analysis of largest eigenvalues and implementation of SOC

**Figure 6:**
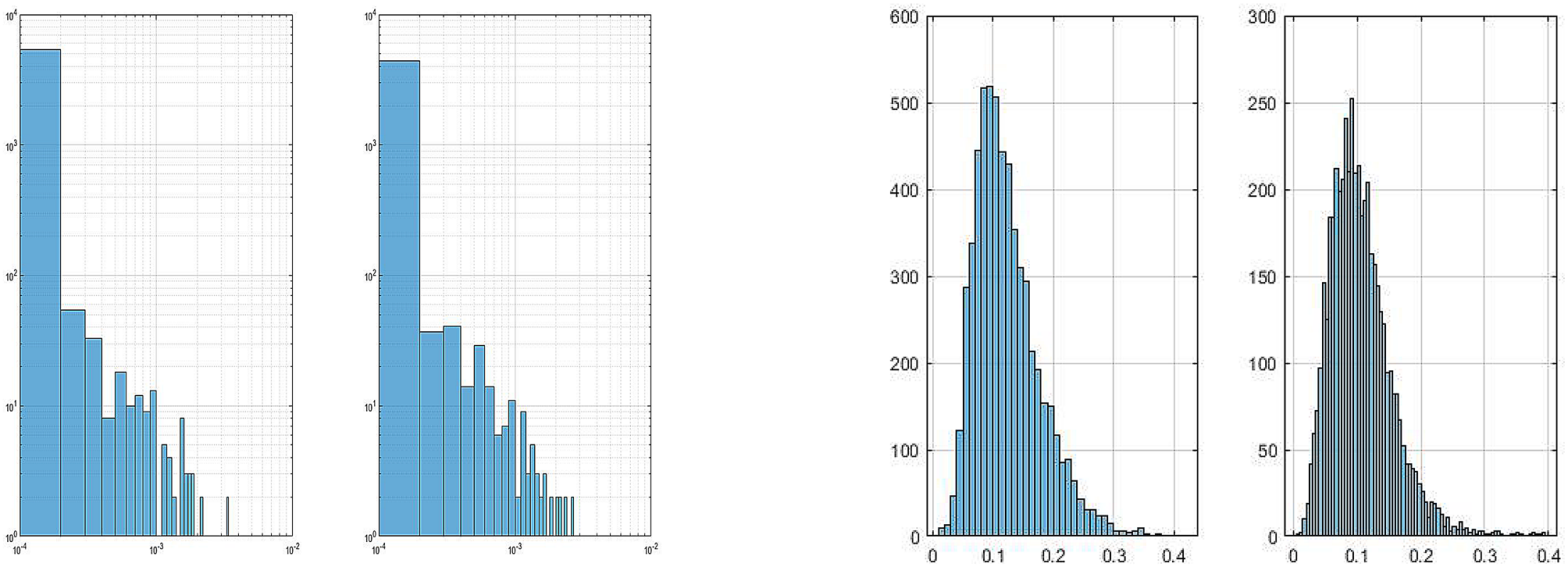
Example of empirical spectral density function of the largest eigenvalues of spatio-temporal tensor expression profile of color variations (left: subject from group C in log-log scale; right: subject from group DM).

**Figure 7:**
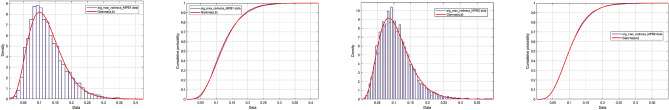
Fitted Gamma probability and cumulative density functions to the tails of the empirical spectral density function of the largest eigenvalue of subjects of group DM.

Examples of $\rho (\lambda _{max})$ of subjects belonging to group C (in log-log scale) and DM are presented in Fig. 6. Furthermore, statistics of the ESD function of the largest eigenvalues of normalized spatio-temporal tensor expression profiles for color variations among subjects of group DM and C are presented in Tables 3 and 4. One can note from the statistics of these tables that the fitted $GP(k,\sigma )$ to group C features positive shape parameter ($k>0$) as opposed to $k<0$ of subjects belonging to group DM. This observation holds for majority of subjects (7 out of 9 in C vs. 15 out of 18 in DM), which is an indication of the feasibility of establishing power law among those subjects of group C, whose tails of their corresponding $\rho (\lambda _{max})$ decay as a polynomial. Furthermore, $\Gamma (\beta ,\theta )$ as an estimator for the Tracy–Widom distribution was fitted to $\rho (\lambda _{max})$ of those subjects from group DM whose *k* in $GP(k,\sigma )$ were negative (15 in DM). This is presented in terms of both fitted PDF and CDF of $\Gamma$ distribution in Fig. 7. Given that these properties do not universally apply to all the subjects of either group C or DM, relying on *sgn*(*k*) as the sole basis for a classifier in an unsupervised learning manner results in a sensitivity of $83.3\%$ and $77.8\%$ for group DM and C, respectively. We can further observe that the magnitude of $\sigma$s of group C are significantly smaller than those of group DM, which if fused with both *sgn*(*k*) and $|k |$, could theoretically outperform the classifier that is based only on *k*.

We observed, however, as exemplified by Table 3, that all those subjects of group C showing $sgn(k)>0$ exhibit $\gamma \in [-1.2,-1.55]$, which according to SOC falls within the range of healthy brain activity. In addition, all subjects of group DM showing $sgn(k)<0$ followed an estimated Tracy–Widom distribution, exhibiting a more random nature of tensor profile.

**Table 3:** Fitted generalized pareto and power law exponent (SOC) to the data of subjects belonging to group C.

	C								
	$C_1$			$C_2$			$C_3$		
	$C_{11}$	$C_{12}$	$C_{13}$	$C_{21}$	$C_{22}$	$C_{23}$	$C_{31}$	$C_{32}$	$C_{33}$
$GP(k,\sigma )$	(0.0869,0.0001)	(0.1013,0.0001)	(0.1697,0.0001)	(0.5628,0.0030)	(0.4382,0.0033)	(1.0249,0.0020)	(1.1599,0.0004)	(0.0897,0.0031)	(0.4650,0.0016)
$\Gamma (\beta ,\theta )$	N/A	N/A	N/A	N/A	N/A	N/A	N/A	N/A	N/A
$\gamma$	$-$ 1.53	$-$ 1.52	$-$ 1.45	$-$ 1.20	$-$ 1.20	$-$ 1.21	$-$ 1.30	$-$ 1.21	$-$ 1.23

$C_{ij}$ is the *j*th video segment of subject *i*.

**Table 4:** Fitted Generalized Pareto and Gamma distribution to the data of subjects belonging to group DM.

	DM								
	$DM_1$			$DM_2$			$DM_3$		
	$DM_{11}$	$DM_{12}$	$DM_{13}$	$DM_{21}$	$DM_{22}$	$DM_{23}$	$DM_{31}$	$DM_{32}$	$DM_{33}$
$GP(k,\sigma )$	($-$ 0.378,0.156)	($-$ 0.334,0.131)	($-$ 0.361,0.127)	($-$ 0.455,0.724)	($-$ 0.135,0.665)	($-$ 0.172,0.758)	($-$ 0.745,0.713)	($-$ 0.811,0.916)	($-$ 0.565,0.748)
$\Gamma (\beta ,\theta )$	(5.449,0.022)	(4.935,0.021)	(4.344,0.023)	(4.948,0.006)	(2.283,0.008)	(1.783,0.015)	(3.310,0.183)	(5.450,0.146)	(3.615,0.133)
$\gamma$	N/A	N/A	N/A	N/A	N/A	N/A	N/A	N/A	N/A

$DM_{ij}$ is the *j*th video segment of subject *i*.

### Classification based on an Ensemble of learners

**Figure 8:**
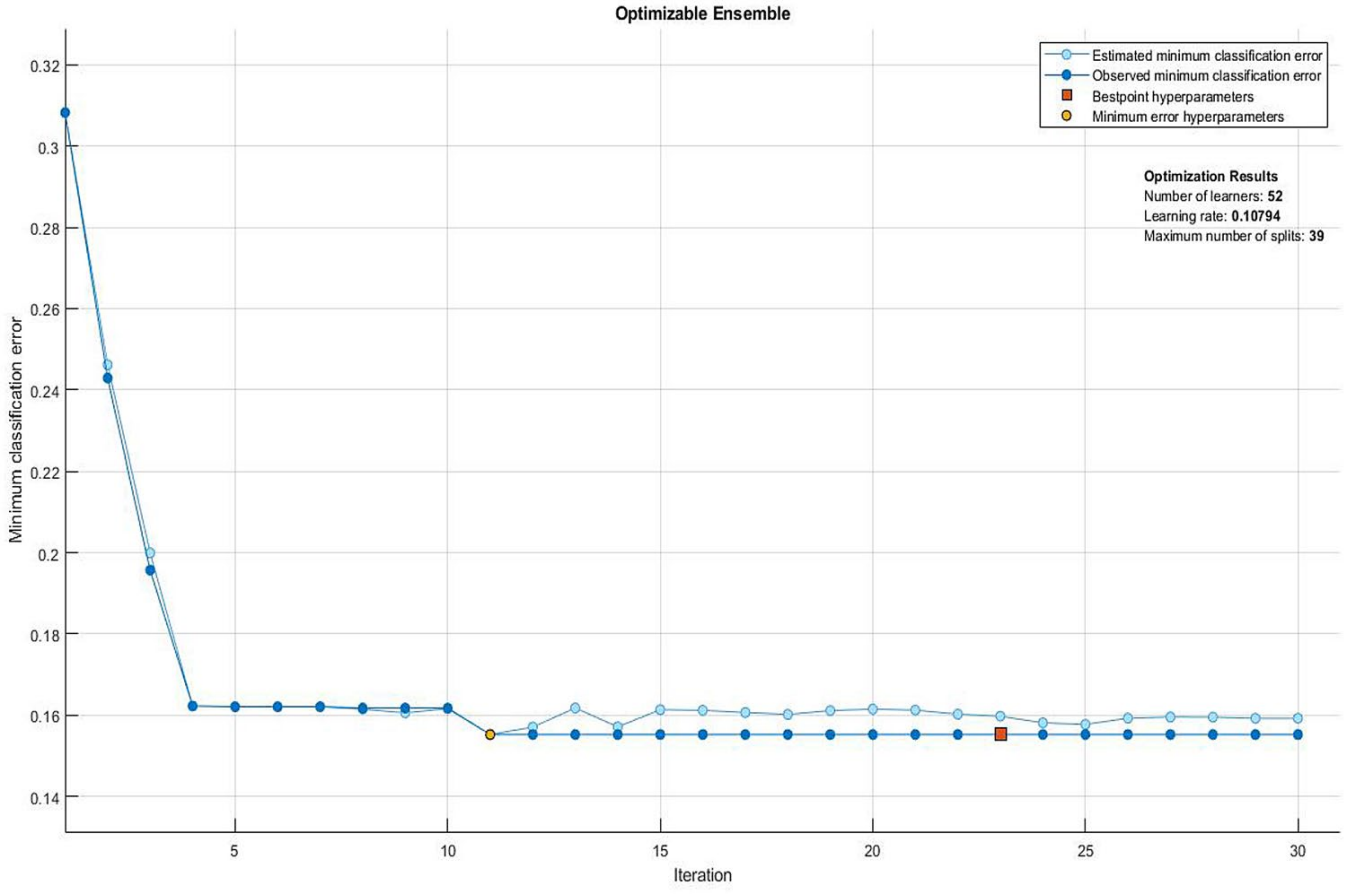
Observed minimum classification error of the best performing ensemble of learners.

**Figure 9:**
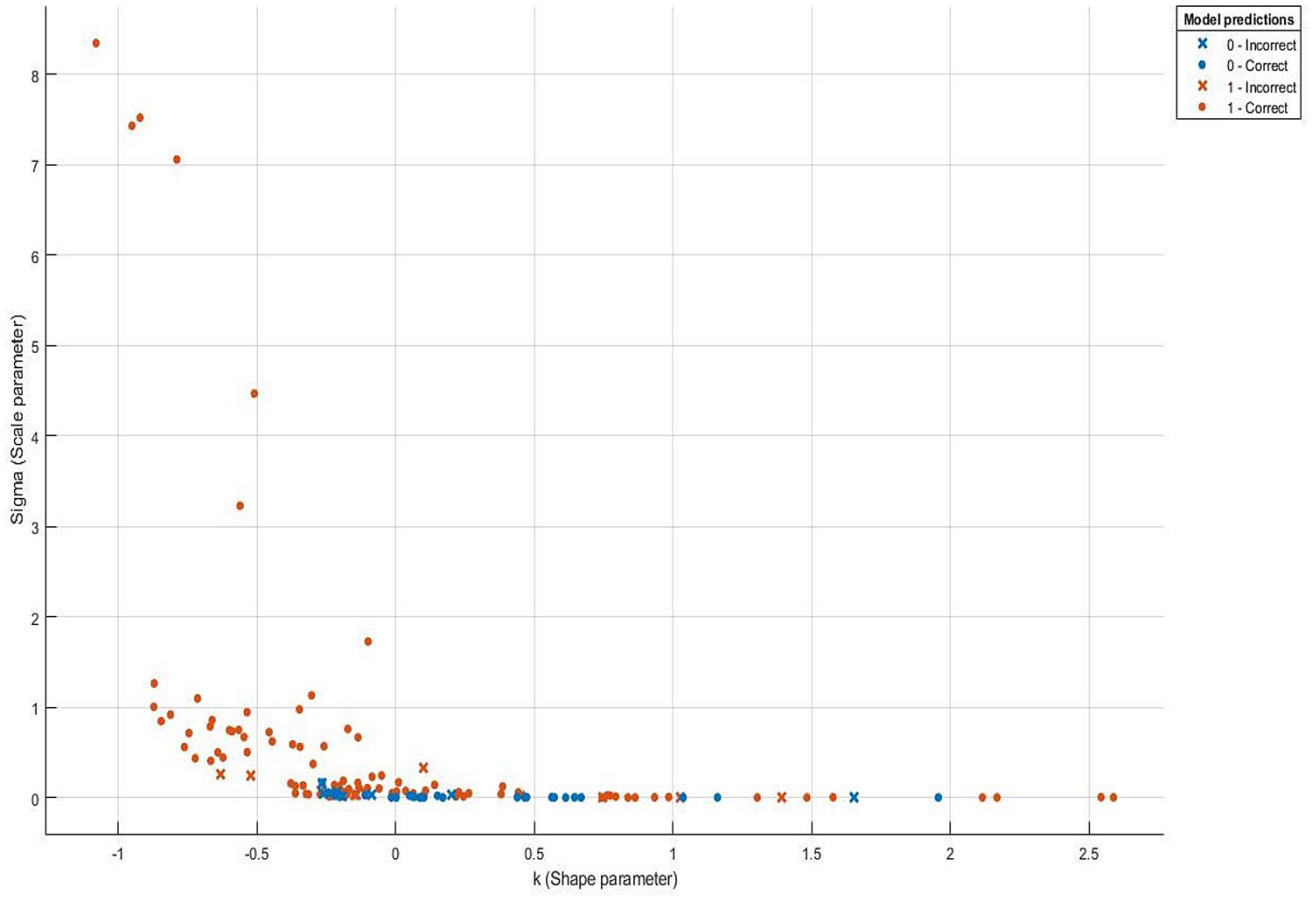
Scatter plot of the ensemble of model predictions.

**Figure 10:**
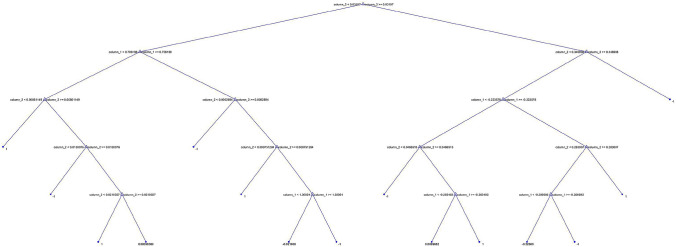
Example of one out of ten decision trees.

**Figure 11:**
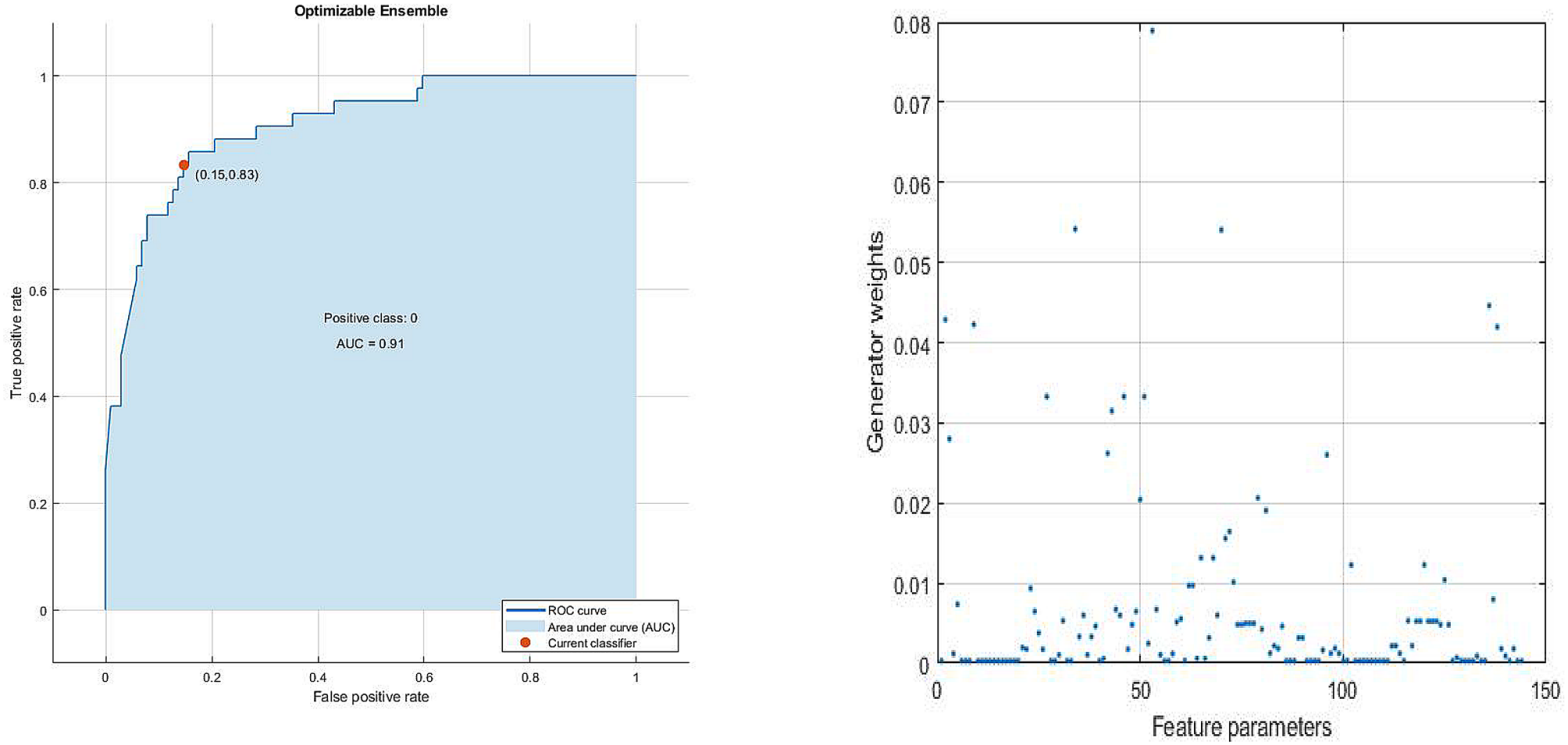
ROC curve (left) and scaled trained weights of our ensemble of learners (right).

Out of all the potential ensembles of learners listed in Fig. 3, the best performance in terms of observed minimum classification error of 0.155 was obtained out of a boosting ensemble of 10 weak and shallow decision tree learners, with an optimal learning rate of 0.469, and maximum split size of 38. Observed minimum classification error of the best performing ensemble of learners and its corresponding scatter plot of model predictions are shown in Figs. 8 and 9, respectively. Furthermore, an example of a decision tree in which the ensemble is based upon is presented in Fig. 10. To obtain optimal prediction model while aggregating predictive learners, all trees were given equal weights, being the most straightforward approach by simply averaging over the pre-tuned base models. The best point hyperparameters were obtained out of 52 learners featuring an optimal learning rate of 0.107. Given that the number of subjects in class DM was twice as that of class C, and in order to cope with class imbalance and skewed nature of the data, RUSBoost with maximum split size of 39 and split criterion of maximum deviance reduction while finding all the surrogate decision splits were applied. To overcome overfitting, fivefold cross-validation was applied. Bayesian optimization was preferred over other techniques, due to gathering observations with highest information while simultaneously incorporating prior beliefs. The performance of the ensemble of learners on the pool of individual video segments, in terms of classification accuracy, sensitivity and specificity reached $86.1\%$, $93.0\%$ and $71.4\%$, where the corresponding ROC curve and scaled trained weights *w* exhibiting a sparse profile are presented by Fig. 11.

**Figure 12:**
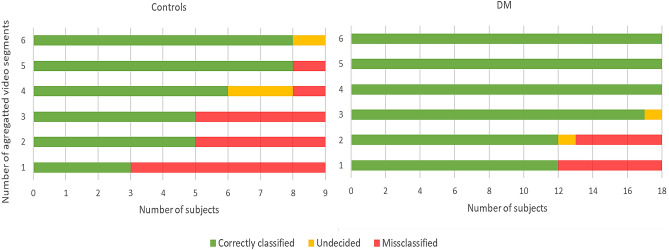
Classification results based on the majority voting of video segments, each column representing the number of aggregated video segments.

The results of final phase of classification process, i.e., fusion of 6 classified video segments of each subject reserved for testing (following the LOO-XV strategy) by applying equally weighted majority voting technique leading to a decision as whether the subject belongs to group C or DM, are presented in Fig. 12. The results show that by integrating classification results from 3 video segments of group DM, only one subject was misclassified (sensitivity of 94.4%) while integration of 4 video segments or more lead to a sensitivity of 100%. Similar results were observed among subjects of group C, in which integration of classification outcomes from 5 video segments resulted in the misclassification of only one subject. Integration of results from 6 video segments shifted the label of the misclassified subject into undecided, due to an equal split in the voting outcome.

## Discussion and concluding remarks

A useful demarcation line that makes the distinction between our proposed POCD and existing solutions crisp and easy to apply, can be formulated as follows. Our POCD is an inexpensive solution that only requires a digital camera and a screen. Given recent advances and surge of interest in smartphones and tablets that are equipped with complex and powerful processors and high resolution peripherals such as illumination systems (LEDs) and cameras, converting our proposed POCD to a home-based solution is feasible. The analysis of collected videos and potential outcomes of such investigations can be performed on a centralized cloud-based server maintained by healthcare professionals. This enables both patient and care givers to automatically update the status of the patient and plan future healthcare actions accordingly. We believe that the same principle could be applied to detect anemia, jaundice and infection and dehydration, using color magnification of lower eyelid mucosa, face and sclera and by observing increased heart rate, respectively. Even though no individual in our study group was diagnosed with obstructive sleep apnoea (OSA); which might result in exhibiting higher levels of haemoglobin, it is our belief that our algorithm would not suffer from this condition, as it relies on the time series associated with the difference between highest and lowest values of color intensity. This results in our technique being independent from the absolute values of haemoglobin, shown to be high in subjects with OSA and low in subjects with anaemia, as the computations are based on relative values of haemoglobin level. Combined with our study on tracking subject’s iris and pupil movements, deformations and dilation, we expect to be able to further detect diabetic encephalopathy.

Even though Eulerian video magnification algorithm does not provide superior performance in terms of computational speed compared to the proposed method in^25^, it has been proven to be a very robust algorithm for revealing subtle changes in both color and motion deformations that fall outside human visual limits. Limitations with regards to video quality metrics, including noise level, video quality and long execution time that are associated with the existing video magnification techniques (such as Eulerian video magnification, phase-based video magnification, Riesz pyramid for fast phase-based video magnification and enhanced Eulerian video magnification) need to be taken into consideration.

The conclusions drawn on the statistics of the ESD function of the largest eigenvalues of normalized spatio-temporal color tensor expression profile, and their tails, were build upon modeling this process as a covariance matrix model in RMT. Even though we observed that in a majority of cases in group C, the tails of these ESD functions decay with a power law exponent mimicking systems exhibiting SOC, while those of group DM follow Tracy–Widom statistics, a natural question of great importance is whether the Information-plus-noise model would be a better choice, which could lead to a concise universal results on the statistics of the tails and their decay rate. It should be also noted that sharp edges in the bulk of these ESD functions were not observed. Another important aspect is the study of subspace stability spanned by the eigenvectors associated with the largest eigenvalues. Following the subspace spanned by these eigenvectors, one expects that the top eigenvector wobble around the true direction either due to the measurement noise or due to the presence of a systematic rotation caused by a hidden mechanism. Having said that, our hypothesis is that there should be a genuine motion of the largest eigenvector in time towards the uniform vector among subjects belonging to group C and away from the uniform vector over time among subjects of group DM. Studying the statistics of the largest eigenvectors and the stability of their corresponding spanned subspace is part of our future work. Our goal is not only to distinguish patients diagnosed with DN-related complications from a control group, but to map the journey, find transition points from mild to severe complications throughout this journey and to stage the disease, by studying different aspects of these eigenvectors.

As part of our future work, we intend to investigate the performance of nested optimization-based algorithms that concurrently tune and find learner’s hyperparameters and the optimal weights, to combine the ensembles of learners on a larger population sample size. The apparent shortcoming of this study is the small sample size and ambiguities around calculation of the power of the study. However, it is worth noting that: (1) in order to make *a priori* power analysis, we would need to have an idea of what the size of the effect will be, and the variance of the variable of interest. Since this is a proof-of-concept study, no reliable estimation was available. (2) Intensity maps presented in Fig. 5 show that the mean of intensity difference among subjects of group DM is approximately 22 times larger than that of group C, which suggests that these two groups are indeed different. Therefore, this result is deemed as statistically meaningful despite study’s small sample size.

Even though our ensemble learning classification technique based on COWE reached a sensitivity of 100% in detecting subjects diagnosed with DN after integrating only 4 video segments (each segment approximately two min long), we believe that recent techniques such as Cross-validated Optimal Weighted Ensemble with Internally Tuned Hyperparameters (COWE-ITH) could outperform our algorithm in terms of required number of video segments. In addition, there is a strong evidence that fusion of information obtained by investigating both variations in skin color and deformations in subject’s iris and pupil movements will outperform the outcome of each individual observation. This paves the road towards the design and development of an inexpensive home-based contact-less, non-invasive diagnostic POCD for early detection of diabetic neuropathy and its associated complications.

## Data Availability

Recorded videos of subjects are available to interested readers.
